# Supplementary material for: Learning curve and surgical outcome of robotic assisted colorectal surgery with ERAS program
Source: Sci Rep. 2022 Nov 29;12:20566. doi: 10.1038/s41598-022-24665-w (PMC9709162; doi:10.1038/s41598-022-24665-w)
Supplement: Supplementary file 1 — Supplementary Information 1. [file 41598_2022_24665_MOESM1_ESM.zip › LSC Raw data-Ñ[▒K/132/N_ñw╝╨░O▒Kñσ.pdf]

W52 / W52

臺中榮民總醫院
手術室護理記錄

[redacted]

基本資料

- 室別： 二醫手術室-31室，手術科別： CRS，手術類別： 常規，日期： 2021/08/11
- 進入時間： 1310 ； 麻醉開始時間： 1315 ； Time Out時間： 1425 ； 劃刀時間： 1426 ； 手術結束時間： 1905 送出時間： 1910 ；
- 麻醉方式： GE
  診斷： S colon ca.
  手術名稱： Laparoscopic Anterior resection and anastomosis - malignant
  手術局部用藥： Nil
  術前預防性抗生素：
  - 抗生素名稱一： Veterin 1GM IVD/由麻醉護理給予
  - 時間： 1410
  - 上次簽章: 林采儀

  ，追加預防性抗生素：
- 手術醫師： 林俊佘
- 麻醉醫師： VS 張思玲
-
  - 刷手護士A ： 彭馨慧，時間： 1310，接替刷手一： 王心俞，時間： 1530，接替刷手二： 葉新娟，時間： 1600
  - 流動護士A ： 林采儀，時間： 1310，接替流動一： 顏育?，時間： 1500，接替流動二： 謝昀安，時間： 1730
- 更多接替及流動護士： 接替刷手三： 顏育?，時間： 1800，接替流動三： 張秋菊，時間： 1800

皮膚狀況

A-V Shunt： 無
術前： 完整
記錄者： 上次簽章: 林采儀
術後： 完整
記錄者： 張秋菊

手術準備

手術臥式 單· ·手術臥式

-
  - 手術臥式： 截石臥式
  - 使用附件： 溫毯 38 ℃，腳架，其它： 大枕頭
  - 皮膚準備： 10%Aq Betadine，Hibiscrub，2% Chlorhexidine
  - 電燒： 使用電燒: 電極片位置： 其它： 床上迴路電極墊，記錄者： 上次簽章: 林采儀

止血帶T： 無

植入物

無

標本送檢

- 細菌培養 無
- 細胞學 無
- 冰凍切片 無
- 病理標本
  組織名稱一： S colon
  ,
  組織名稱二： IMA root
  ,
  組織名稱三： distal cut end
- 截肢 無

標本交付 標本： 三 袋 ，標本： 零 盒 ，標本： 零 瓶 ，細胞學： 零 管 ，Culture Tube： 零 管 ，被交付醫師：Fellow 陳映菁，

- 記錄者： 張秋菊
- 時間： 1900

傷口術後敷料

傷口敷料：

- 請於圖上標示部位：

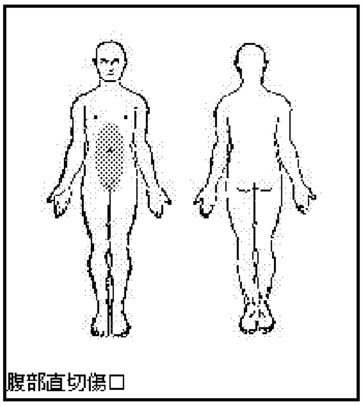

- 傷口敷料： 紗布＋mepore

導管

導管	天數	導管資訊	插入日期	插入時間	更換日期	拔除日期	拔除時間	拔除原因	預計拔除/更換	預計拔除/更換日期
Penrose Drain(1)	1	號碼:12 mm.; 部位:骨盆腔; 註記:右腹;	2021/08/11	1740						
A-line (1)	1	號碼:20 Fr; 部位:左手;	2021/08/11	1420						
Double-J(1)	1	號碼:6 Fr.; 部位:左側輸尿管;	2021/08/11	1830						
Foley(1)	1	號碼:18 Fr.; 部位:膀胱;	2021/08/11	1850						

用物計數

- 手術中出血量： 100 c.c.
- 手術中尿量： 500 c.c.
-
    - 其它： 無

用物計數： 單一切口

- 1.器械： 正確，2.縫針： 正確，3.刀片： 正確，4.紗布： 正確，5.腹部墊： 正確，6.紗球： 未使用，7.PEANUTS ： 未使用，8.紗條： 未使用，9.絲帶： 未使用，10.針頭： 未使用，11.Vessel loop： 未使用，12.Boots： 未使用，13.Jelco： 未使用，14.棉條： 未使用，
    - 第一次：
        - 刷手護士 ： 葉新娟
        - 流動護士： 上次簽章: 謝昀安
        - 時間： 1740

  ,
    - 第二次：
        - 刷手護士 ： 葉新娟
        - 流動護士： 上次簽章: 謝昀安
        - 時間： 1750

  ,
    - 第三次：
        - 刷手護士 ： 顏育?
        - 流動護士： 張秋菊
        - 時間： 1830

備註

備註： NIL

- 記錄者： 張秋菊
- 時間： 1905

填寫護理人員 張秋菊

正式摘要

W52 / W52

doctorRank:B
